# A Modified Protocol for Bisulfite Genomic Sequencing of Difficult Samples

**DOI:** 10.1007/s12575-009-9010-3

**Published:** 2009-06-24

**Authors:** Jane J Pappas, André Toulouse, WEC Bradley

**Affiliations:** 1 Centre de Recherche du C.H.U.M., Université de Montréal, Montreal, Canada; 2 Division of Experimental Medicine, McGill University, Montreal, Canada; 3 Department of Anatomy/Neuroscience, University College Cork, Cork, Ireland

**Keywords:** bisulfite genomic sequencing, multiple restriction enzyme digestion, methylation

## Abstract

The bisulfite genomic sequencing protocol is a widely used method for analyzing DNA methylation. It relies on the deamination of unmethylated cytosine residues to uracil; however, its high rates of DNA degradation and incomplete cytosine to uracil conversion often lead to failed experiments, uninformative results, and false positives. Here, we report the addition of a single-step multiple restriction enzyme digestion (MRED) designed to differentially digest polymerase chain reaction products amplified from unconverted DNA while leaving those of converted DNA intact. We show that for our model system, *RARB2* P2 promoter, use of MRED increased informative sequencings ninefold, and MRED did not alter the clonal representation in one fully methylated cell line, H-596, treated or not with 5-azadeoxycytidine, a methylation inhibitor. We believe that this method may easily be adapted for analyzing other genes and provide guidelines for selecting the most appropriate MRED restriction enzymes.

## 1. Introduction

The bisulfite genomic sequencing (BGS) protocol [1,2] is a method of choice for analyzing DNA methylation at the nucleotide level. Sodium bisulfite is used to convert unmethylated cytosine residues to uracil residues in single-stranded DNA. In particular, bisulfite conversion consists of three sequential chemical reactions: sulfonation of cytosine to cytosine-6-sulfonate, deamination to uracil-6-sulfonate, and desulfonation to uracil. However, since 5-methylcytosine residues are nonreactive, they remain intact. The bisulfite-converted DNA is then amplified with specific primers designed for converted DNA, and purified polymerase chain reaction (PCR) products, which are usually subcloned, are sequenced.

Bisulfite conversion is so powerful that it has been paired with numerous techniques other than traditional sequencing, including: methylation-specific PCR [3], combined bisulfite restriction enzyme analysis [4], methylation-sensitive single nucleotide primer extension [5], methylation-sensitive single-strand conformation analysis [6], MethyLight [7], oligonucleotide microarray methods [8], denaturing high-performance liquid chromatography with bisulfite genomic sequencing [9], pyrosequencing methylation analysis [10], and methylation-sensitive high-resolution melting-curve analysis [11], among others (*see *[12] for a review). In addition, many methylation analysis kits are also commercially available.

Unfortunately, high rates of DNA degradation and incomplete conversion reactions often lead to decreased efficiency of the assay. Many attempts have been made to minimize template degradation and/or maximize cytosine conversion [13-19], but overall, the bisulfite conversion protocol has remained unchanged, and no other high resolution or positive display methylation analysis protocol exists. As a result, the BGS protocol, as well as any technique paired with the bisulfite conversion reaction (and, hence, founded on the assumption that conversion is complete) often generate few or no informative results.

In our studies of the *RARB2* P2 promoter [20], we found that incomplete conversion was an insurmountable challenge even after modifying the protocol in numerous ways. We, therefore, aimed to circumvent these issues altogether by depleting the PCR populations of products amplified from partially converted or unconverted DNA using a multiple restriction enzyme digestion (MRED) approach. We found that informative sequencings were increased ninefold using it. We believe that this method may easily be adapted for analyzing the detailed methylation status of other genes presenting incomplete cytosine to uracil conversion, and we provide guidelines for selecting the most appropriate restriction enzymes (REs).

## 2. Materials and Methods

### 2.1. Cell Culture and Genomic DNA Extraction

#### 2.1.1. Cell-Line Provenance

Twenty-one cell lines were cultured. CALU-1, SK-MES, CACO-2, COLO-201, COLO-205, HCT-15, and LS-180 were obtained from the American Type Culture Collection (Rockville, MD). The CALU-1 daughter cell lines, C-19 and C-59, are *RARB2*-transfectants that were established in our laboratory [21]. MM-1 was also established in our laboratory [6]. NCI-H23, NCI-H82, NCI-H125, NCI-H157, NCI-H520, and NCI-H596 were supplied by Dr. Adi Gazdar (NCI, NIH, Bethesda, MD). NBE-E_6_E_7 _[22] was provided by Dr. Jean Viallet (Gemin X Biotechnologies Inc., Montreal, Québec). SW 1222 was given to us by Dr. Clifford Stanners (McGill University, Montreal, Québec). Qu-DB was provided by Dr. Barbara Campling (Queen's University, Kingston, Ontario). T47D, MDA-MB-231 (MB-231), ZR-75B, and HS-578T were kindly provided by Dr. Morag Park (McGill University, Montreal, Québec).

#### 2.1.2. Cell Culture

CALU-1, CACO-2, SW-1222, and LS-180 were grown in α-MEM medium (Invitrogen, Carlsbad, CA) supplemented with 10% heat-inactivated fetal calf serum (FCS; Wisent Bioproducts, Saint-Jean-Baptiste de Rouville, Québec). NBE-E_6_E_7_ was grown in keratinocyte-serum free medium (Invitrogen), supplemented with 50 μg/ml bovine pituitary extract, and 5 ng/ml recombinant human epidermal growth factor (Invitrogen). All other cells were grown in RPMI-1640 medium (Invitrogen) supplemented either with 5% (SK-MES, NCI-H23, NCI-H125, NCI-H520, Qu-DB, and HS-578T) or 10% FCS (NCI-H82, NCI-H157, MM-1, T47D, MDA-MB-231, ZR-75B, COLO-201, COLO-205, and HCT-15). Where indicated, cells were treated with 1 μM 5-azadeoxycytidine.

#### 2.1.3. Genomic DNA Extraction

**Figure 1:**
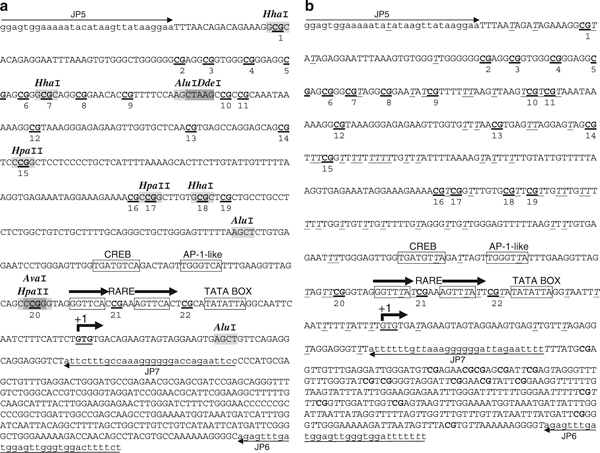
***RARB2* sequence under analysis**. **A** Unconverted sequence; **B** converted sequence (all non-CpG-cytosines have been replaced with thymidines). The 541 bp sequence analyzed is comprised between oligonucleotides JP5 and JP7. **CG** = CpG dinucleotide under investigation (*n* = 22); *T* = non-CpG-cytosine converted to thymidine following bisulfite treatment (*n* = 82); *boxes* promoter elements; *gray-shaded sequences* RE sites (please note that these sites are absent in the converted sequence); *+1* transcription start site. Direct repeats of the RARE are indicated. The oligonucleotide sequences are *underlined* and are designed for converted DNA.

Genomic DNA was extracted using the standard phenol-chloroform technique followed by proteinase K treatment to ensure complete protein removal [23]. DNA was then digested with the *Pst*I RE (New England BioLabs, Ipswich, MA) according to the supplier's directives to shorten the fragment (2.95 kb) containing the target *RARB2* P2 promoter sequence investigated (541 bp; Figure 1), thereby reducing the possibility for regional double-strand formation [24]. *Pst*I was the only RE available for the sequence under analysis.

### 2.2. Bisulfite Conversion

#### 2.2.1. Bisulfite Conversion

Multiple DNA samples of each cell line, 1–2 μg each, were treated with bisulfite as per the BGS protocol [1], with minor modifications. Briefly, *Pst*I-digested genomic DNA (1–2 μg) was precipitated and resuspended in 25 μl distilled water. DNA was denatured with 0.3 N NaOH at 37°C for 15 min. Fifteen microliters of freshly prepared 10 mM hydroquinone (Sigma–Aldrich Canada Ltd., Oakville, Ontario) was added to the tubes while at 37°C, and solutions were carefully mixed by inversion with minimal aeration. Two hundred fifty microliters of freshly prepared 3.6 M sodium bisulfite (Sigma–Aldrich), pH 5.0, was added to the tubes while at 37°C, and solutions were again carefully mixed by inversion with minimal aeration. Reaction volumes were overlaid with mineral oil and incubated at 55°C for 16 h in the dark. Aqueous phases were transferred to new tubes and desalted with Wizard Magic Miniprep DNA purification resins (Promega, Madison, WI). DNA was eluted with 120 μl distilled water and residual alcohol was removed using speedvac centrifugation. Ten microliters 3N NaOH was added to the remaining 100 μl and allowed to incubate for 15 min at 37°C. DNA was precipitated with 33 μl 10 M sodium acetate pH 7.8 and 300 μl chilled ethanol using glycogen as a carrier in an ice-water bath for 10 min and then centrifuged at 13,000×*g* for 60 min. The precipitate was resuspended in 100 μl TE pH 8.0.

#### 2.2.2. PCR Amplification

A 541-bp sequence containing 22 CpG dinucleotides (Figure 1A) was identified within the human *RARB* P2 promoter [20]. PCR amplification consisted of two rounds of amplification: round 1 primers consisted of the upper primer JP5 (5'-GGAGTGGAAAAATATATAAGTTATAAGGAA-3') and the lower primer JP6 (5'-AAAAAAATCCACCCAACTCCATCAAACTCT-3'); round 2 semi-nested primers consisted of JP5 and the lower primer JP7 (5'-AAAATTCTAATCCCCCCTTTAACAAAAAAT-3'). Cycling conditions were: 94°C/4 min × 1 cycle; 94°C/1 min, 61°C/2 min, 72°C/2 min × 5 cycles; 94°C/1 min, 61°C/1.5 min, 72°C/1.5–2 min × 25 cycles; 72°C/5 min × 1 cycle. Primers were designed following the guidelines found in [25]. In particular, they were designed not to contain CpG dinucleotides so that PCR amplifications were not biased according to methylation status. The minimum number of non-CpG-cytosines available for measuring the rate of cytosine to uracil conversion, for quality control assessment, is 74. This does not include the one non-CpG-cytosine within the region complementary to JP5 and the seven non-CpG-cytosines within the region complementary to JP7 (*see* section 2.3.2, Special Considerations).

### 2.3. MRED

#### 2.3.1. Restriction Enzyme Selection

The original (Figure 1A) and converted (Figure 1B) sequences were entered into NEBcutter V.2 at http://tools.neb.com/NEBcutter2/index.php (New England BioLabs), and RE maps and lists were made. Potential MRED isoschizomers were screened based on the following criteria: (1) RE sites should selectively cut unconverted DNA while leaving converted DNA intact; (2) RE sites may or may not contain CpG-cytosines but should contain at least one non-CpG-cytosine; (3) if RE sites do not contain at least one non-CpG-cytosine, then RE sites should not contain either of the following: (a) a CpG-cytosine, (b) a 3'-C if immediately followed by a G within the downstream sequence, or (c) a 5'-G if immediately preceded by a C within the upstream sequence (for a summary of these criteria, *see* Table 1).

**Table 1:** Guidelines for choosing (MRED) restriction enzymes

1	The RE site must contain at least one non-CpG-cytosine
2	The RE site must be abolished following cytosine to uracil conversion
3	The most 5' and 3' nucleotides of the sequence must not form CpG dinucleotides with upstream or downstream sequences respectively
4	The RE site preferably does not have a site within the primer sequences
5	The RE site may be cut with a methylation-sensitive RE, since PCR products are not methylated

#### 2.3.2. Special Considerations

(1) Since primers can anneal to DNA sequences with less than perfect complementarity and since this may potentially involve primer adenines annealing to unconverted non-CpG-cytosines, we omitted all MRED enzymes with sites within primer sequences. We reasoned that not omiting them might incorporate some incompletely converted molecules. (2) It may be noteworthy to clarify that methylation-sensitive RE may indeed be used since the DNA being cleaved is synthesized in vitro (via PCR) and, hence, not methylated. (3) The five RE we chose (*Alu*I, *Ava*I, *Dde*I, *Hha*I, and *Hpa*II) have a total of 11 sites within the target sequence, and each RE contains one non-CpG-cytosine, except *Ava*I, which contains two. Since potential causes for lack of single-strandedness (incomplete denaturation, reannealing of complementary strands, or formation of secondary structures between complementary segments within a same strand) can occur anywhere within the entire sequence, we selected a group of enzymes having sites more or less evenly distributed across the entire sequence. RE sites are shown in Figure 1A, and their characteristics are listed in Table 2. (4) REs with star activity should be avoided. None of the enzymes chosen here have star activity, and conditions that are known to potentially cause star activity in certain REs (including high levels of glycerol or Mn2+, low or high pH, low or high ionic strength, or presence of DMSO or 2-mercaptoethanol) were also avoided.

**Table 2:** Characteristics of the MRED restriction enzymes used for *RARB2*

RE	Site	**No. of non-CpG-C within RE site**^**A**^	**No. of sites within sequence**^**B**^	A*B
AluI	AG**C**T	1	3	3
AvaI	**CC**CGGG	2	1	2
DdeI	**C**TNAG	1	1	1
HhaI	GCG**C**	1	3	3
HpaII	**C**CGG	1	3	3
Total				12

CpGs are underlined. Bisulfite-convertible cytosines are in boldface. Please note that these cytosines are thymidines in the PCR-amplified product representing bisulfite converted DNA and are therefore not recognized by the RE in question

#### 2.3.3. Multiple Restriction Enzyme Digestion

Eighty microliters (~2 μg) of PCR products were digested with 10–20 units each, *Alu*I, *Ava*I, *Dde*I, *Hha*I, and *Hpa*II (New England BioLabs) as per the supplier's directives for 2.5 to 4 h.

### 2.4. Gel Extraction and Subcloning

MRED digestions were ethanol precipitated, resuspended in TE buffer pH 8.0, and electrophoresed on 3% agarose gels. Undigested products (the 541-bp band) were precisely excised using a new scalpel blade and extracted using the Sephaglas™ BandPrep Kit (GE Healthcare, Uppsala, Sweden). Gel extracted products were subcloned into pBluescript (Stratagene, La Jolla, CA) or pCR2.1 (Invitrogen, Carlsbad, CA) vectors using T4 DNA ligase (NEB) and transformed into competent DH5α *Escherichia coli* cells (Invitrogen).

### 2.5. Sequencing

Plasmid DNA was purified with Qiagen Maxi or Midi kits (Qiagen, Valencia, CA) and sequenced using universal T3 and/or T7 primers. Sequencings were performed in-house or at BioS&T, Inc., Montreal, Canada.

#### 2.5.1. Special Consideration

Each sample was derived from an independent bisulfite-treated DNA sample (i.e., only one bacterial colony was sequenced per bisulfite reaction to ensure that sequencings were not derived from the same PCR DNA template).

## 3. Results and Discussion

**Figure 2:**
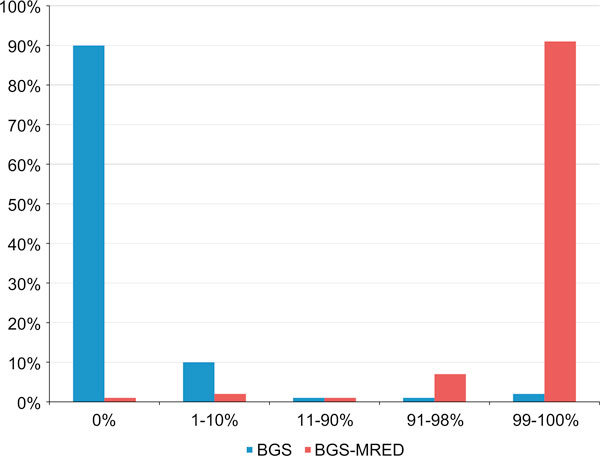
**Rates of nucleotide conversion using the original or the modified protocols**. The conversion status of 74 non-CpG-cytosine residues of the *RARB2* P2 promoter was analyzed following conversion using the standard BGS protocol (blue bars) or the MRED modification (*red bars*). Clones were sequenced and grouped according to the percentage of converted cytosine residues. Results show an increase in the frequency of cytosine to uracil conversion using our modified protocol. While only 10% of samples sequenced using the original protocol (*n* = 200) achieved 99% conversion of non-CpG-cytosines, 91% of samples sequenced using the modified protocol (*n* = 176) achieved 99% conversion. The threshold for the status of informativity was randomly set to 73/74 (99%) conversions to uracil, and this threshold was used to distinguish fully converted sequencings from partially converted ones.

In order to compare the efficiencies of the original and the modified protocols, we investigated the rates of conversion of the non-CpG-cytosine residues within the *RARB2* P2 promoter region under analysis. These sites are not normally methylated and are, therefore, expected to be fully converted. There are 74 non-CpG-cytosine residues within this region (excluding those found within regions complementary to primers JP5 and JP7): we randomly set the threshold for the status of informativity to 73/74 (99%) conversions to uracil and used this threshold to distinguish fully converted sequencings from partially converted ones. In particular, for a sample to be labeled as fully converted, it must have reached ≥99% conversion of these non-CpG-cytosines. Upon comparison, we found that there was a dramatic increase in the number of informative sequencings using our modified protocol: while only 10% of samples sequenced using the original protocol (*n* = 200) achieved 99% conversion of non-CpG-cytosines, 91% of samples sequenced using the modified protocol (*n* = 176) achieved 99% conversion (Figure 2). It is interesting to note that the majority of the remaining sequencings using the modified protocol were nearly fully converted (91–98%). In contrast, nearly all sequencings using the original protocol were nearly fully unconverted (0% and 1–10%).

**Figure 3:**
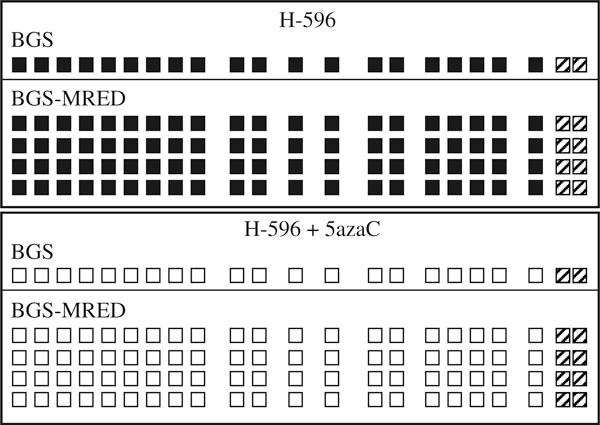
**Schematic representation of the methylation analyses at the 22 CpG sites within the *RARB2* P2 promoter in H-596 cells**. *Top* DNA from untreated H-596 cells; *Bottom* H-596 cells following 5-azadeoxycytidine treatment (fully demethylated). Each sample was derived from an independently bisulfite-treated DNA sample (only one bacterial colony is sequenced per bisulfite reaction to ensure that sequencings are not derived from the same PCR DNA template). *Boxes* represent CpG sites. *Solid box* methylated; *empty box* not methylated; *hatched box* status unavailable.

The use of MRED (using *Alu*I, *Ava*I, *Dde*I, *Hha*I, and *Hpa*II) was shown to prevent methylation bias since both methylated and unmethylated CpG-cytosines were found to be represented at all 22 CpGs in the samples analyzed (*n* = 176; results not shown). In order to determine whether or not there was a bias introduced by the MRED modification, particularly the CpGs contained within the RE sites (1, 7, 15, 17, 18, 20 in Figure 1), we applied MRED to DNA samples previously analyzed using BGS alone. DNA samples previously extracted from H-596 lung adenosquamous carcinoma cells treated or not with 5-azadeoxycytidine, a methylation inhibitor. Using BGS, they were found to be fully methylated from untreated cells or fully unmethylated following treatment with 5-azadeoxycytidine (Figure 3). When they were resequenced using the modified protocol, the results were identical: 8/8 sequencings displayed complete methylation (Figure 3, top) or complete demethylation (Figure 3, bottom) at all 20 informative sites. This clearly demonstrates that the introduction of the MRED step does not introduce a bias at any of the 20 informative sites.

Using the BGS protocol [1] in over 400 sequencings, even with some modifications, we found that incomplete cytosine to uracil conversion and DNA degradation formed insurmountable challenges. In a first effort, numerous attempts to reduce the rate of target DNA degradation were made, including: (1) increasing the amount of genomic DNA from 1 to 10 μg; (2) decreasing the duration of the bisulfite conversion reaction [15]; (3) incorporating up to 5 μg salmon sperm DNA; or (4) using agarose beads [16]. None of these modifications improved the rate of DNA degradation. In addition, the absence of PCR products could not be associated with any one factor (it was variable and unpredictable).

In a second effort, numerous attempts to increase the rate of cytosine to uracil conversion were made, including: (1) digesting the genomic DNA with an endonuclease such as *Pst*I to create smaller fragments containing the target sequence thereby reducing the possibility for regional double strand formation [24]; (2) denaturing the genomic DNA in an alkaline solution prior to BGS treatment, thereby beginning the BGS protocol with fully denatured DNA; (3) incubating the bisulfite reactions at 95°C [24] every 3 h, thereby aiming to maintain complete DNA denaturation; (4) reducing the DNA quantity to as little as 100 ng [13]; (5) increasing the sodium bisulfite concentration (6 M) [15], (6) using high-speed BGS (9 M sodium bisulfite for 20 min at 90°C or 40 min at 70°C) [19], (7) using a lower incubation temperature, such as 50°C [15], to increase the extent of cytosine conversion and/or to reduce the annealing of single-stranded DNA sequences during treatment; (8) monitoring the pH of the solutions to prevent incomplete desulfonation of pyrimidine residues, which may inhibit DNA polymerases, leading to unsuccessful PCR amplifications [12]; (9) changing PCR extension time; (10) annealing temperature; (11) MgCl_2_ concentration; (12) adding dimethyl sulfoxide to inhibit secondary structure formation [26]. In all cases, PCR amplifications were once again unpredictable, and when they were productive, none of these modifications increased the rate of occurrence of fully converted samples following BGS above 0–10%. Different primers were also designed, including fully nested as opposed to seminested primers as was the case here, to no avail.

Such resistance to deamination is a frequent characteristic of *RARB2* (unpublished observation, Dr. Michael Trus, Juravinski Cancer Center, Hamilton, Ontario) and is not unique to *RARB2* (*e.g*., [27-29]). Although high GC content has previously been suggested to cause incomplete conversion [30], the 541-bp region we targeted has a GC content of 51%, 4% lower than that characterizing most CpG islands or promoters [31].

*RARB2* DNA methylation has been shown to be correlated with *RARB2* gene inactivation [32-38], and treatment with a methyltransferase inhibitor, 5-azadeoxycytidine, has been shown to be correlated with demethylation of exonic sequences and reactivation of gene expression [32,34,37,39]. However, few studies have analyzed the detailed methylation pattern of the promoter region [33,40-42,42], and to our knowledge, studies have not analyzed isolated alleles by sequencing only one subclone per bisulfite conversion reaction (*see ***Section** 2.5.1). The vast majority of studies have used methylation-specific PCR, pooling potentially mixed populations of alleles together, as previously described in ref. [38], and not allowing the direct assessment of cytosine to uracil conversion.

To our knowledge, this is the first report of a RE-based method to improve the BGS protocol. This modified protocol is not related to techniques in which RE digestion is used to reveal and/or quantify DNA methylation-dependent sequence differences in PCR-amplified bisulfite-treated DNA [43] or with techniques in which methylation-dependent retention of preexisting sites, such as *Bst*UI (CGCG; following bisulfite-induced sequence conversion), are exploited to quantify DNA methylation at specific loci, such as in the combined bisulfite restriction analysis [4]. These techniques focus on specific CpG sites and are based on the assumption that conversion is complete. In contrast, the present protocol was designed to retain the fine resolution analysis capability of the original BGS protocol. It does so by digesting incompletely converted DNA molecules in the resulting mixed PCR population. Conversion efficiency is not assumed to be 100% but rather is measured directly for every sample.

We hope that studies requiring fine resolution methylation analyses, such as those investigating the various allelic populations within a cell sample and those in which BGS-associated degradation and inefficient conversion impede research progress, will benefit from using this modified protocol, especially given the growing need for protocols capable of interrogating the methylation status at the nucleotide level (e.g., allele-specific methylation) and the growing interest in protocols providing internal quality control parameters. The guidelines for selecting REs are straightforward and may be used for the methylation analysis of any gene. This method requires the addition of only one step, MRED, to the original protocol, adding only 4 h to the 3-day BGS process. While RE selection may be time consuming for some sequences, the same combination of RE may be used for all subsequent sequencings.

## Competing Interests Statement

The authors declare that they have no competing interests.
